# Metabolism Is a Key Regulator of Induced Pluripotent Stem Cell Reprogramming

**DOI:** 10.1155/2019/7360121

**Published:** 2019-05-05

**Authors:** James Spyrou, David K. Gardner, Alexandra J. Harvey

**Affiliations:** School of BioSciences, The University of Melbourne, Parkville, VIC 3010, Australia

## Abstract

Reprogramming to pluripotency involves drastic restructuring of both metabolism and the epigenome. However, induced pluripotent stem cells (iPSC) retain transcriptional memory, epigenetic memory, and metabolic memory from their somatic cells of origin and acquire aberrant characteristics distinct from either other pluripotent cells or parental cells, reflecting incomplete reprogramming. As a critical link between the microenvironment and regulation of the epigenome, nutrient availability likely plays a significant role in the retention of somatic cell memory by iPSC. Significantly, relative nutrient availability impacts iPSC reprogramming efficiency, epigenetic regulation and cell fate, and differentially alters their ability to respond to physiological stimuli. The significance of metabolites during the reprogramming process is central to further elucidating how iPSC retain somatic cell characteristics and optimising culture conditions to generate iPSC with physiological phenotypes to ensure their reliable use in basic research and clinical applications. This review serves to integrate studies on iPSC reprogramming, memory retention and metabolism, and identifies areas in which current knowledge is limited.

## 1. Introduction

The exogenous expression of the transcription factors OCT4, SOX2, KLF4, and c-MYC in both mouse and human somatic cells has enabled the derivation of cells with embryonic stem cell (ESC) -like properties, termed induced pluripotent stem cells (iPSC) [1, 2]. While these reprogrammed cells are capable of self-renewal, demonstrate *in vitro* differentiation potential equivalent to that of ESC and, in mice, are able to contribute to viable chimeras [3], several studies have raised concerns that iPSC retain somatic cell memory and acquire characteristics that may bias cell fate or impair cell function post-differentiation. As iPSC have the capacity to differentiate into cells of each of the three primary germ layers: endoderm, mesoderm, and ectoderm [4], they possess immense potential for clinical applications in disease modelling, drug discovery, and regenerative medicine. It is therefore of great importance for iPSC to be able to appropriately respond to their environment and acquire an ESC-like physiology to ensure that they can be safely and reliably used in the clinic and recapitulate the physiology of disease models in drug discovery and basic research.

Culture conditions and nutrient availability not only affect reprogramming itself but have a long-term impact on the resultant physiology of iPSC. This review therefore discusses recent advances in our understanding of factors that influence the efficiency of the reprogramming process, metabolic restructuring, and retention of somatic cell memory, as well as how it is essential to further elucidate how somatic cell memory is retained for the subsequent optimisation of the reprogramming process to generate iPSC with a physiological ESC-like phenotype and ensure long-term cellular health.

## 2. Reprogramming Necessitates Transcriptional, Epigenetic, and Metabolic Restructuring

In contrast to most somatic cells, which primarily utilise oxidative phosphorylation (OxPhos) for energy production [5], iPSC instead rely primarily on glycolysis [6–8]. This curious metabolic phenotype resembles that of ESC [9] and recapitulates that of the inner cell mass (ICM) of the blastocyst, which is almost exclusively glycolytic [10, 11]. This metabolism is characterised by a high glucose to lactate flux even in the presence of adequate oxygen, a phenomenon known as aerobic glycolysis, first characterised by Warburg [12, 13]. While glycolysis is not as efficient as OxPhos in terms of the number of adenosine triphosphate (ATP) molecules produced per mol of glucose consumed, glycolysis can produce an equivalent amount of ATP in the same duration of time given a high glucose to lactate flux [14]. Glycolysis consequently plays a key role in the production of biosynthetic precursors, such as phospholipids and glycoproteins [15, 16], necessary to support proliferation and regulate cell function, and likely ensures protection of the genome from oxidative stress caused by excessive production of reactive oxygen species (ROS) [17].

Reprogramming to pluripotency involves a transition from a primarily oxidative to a primarily glycolytic metabolic phenotype [6, 9, 18], and this metabolic restructuring takes place in the initial phase of the reprogramming process. Oxygen consumption and ATP production, as well as gene expression levels of pathways such as glycolysis, the pentose phosphate pathway (PPP) and the tricarboxylic acid (TCA) cycle, are remodelled during reprogramming to levels similar to those found in ESC [9, 19, 20]. Following the restructuring of metabolism, the promoters of pluripotent genes undergo DNA demethylation, while those of somatic genes are methylated [21]. This results in the upregulation of endogenous NANOG, OCT4, and SOX2, activating the transcription factor network responsible for the establishment and maintenance of pluripotency [22]. The chromatin structure [23] and the epigenetic landscape [24] are remodelled to resemble those of ESC, to enable ongoing transcription of genes that underpin pluripotency. In addition, telomerase is upregulated [25], with a concomitant lengthening of telomeres to ESC-like lengths [26], providing improved genomic stability and protection against DNA damage. Significantly, as metabolic changes precede the upregulation of pluripotency markers [6], this illustrates that metabolic restructuring is a prerequisite for the successful establishment and maintenance of pluripotency, and that perturbations in this restructuring may have downstream effects on the subsequent stages of the reprogramming process, including remodelling of the epigenome and the successful establishment of a pluripotent state. Equally, altering relative metabolite availabilities during reprogramming, by modulating metabolism, will plausibly impact both metabolic and epigenetic remodelling and hence the acquisition of pluripotency. While the effects of some specific metabolites on improving or reducing reprogramming efficiency have been assessed, understanding how reprogramming under such conditions affects the restructuring of metabolism and the physiology and metabolic phenotype of resultant iPSC is limited.

## 3. Metabolism as a Driver of Reprogramming

A role for metabolism in regulating the acquisition of pluripotency has been demonstrated by studies investigating the effect of promoting glycolysis during the reprogramming process on reprogramming efficiency. Culturing adult human fibroblasts under physiological (5%) oxygen or supplementing culture medium during reprogramming with D-fructose-6-phosphate (F6P), a glycolytic stimulator and intermediate, significantly increases the number of derived iPSC colonies [7, 27]. As both physiological oxygen and F6P promote lactate production [10, 28, 29], they plausibly improve reprogramming efficiency by supporting and facilitating the transition to a primarily glycolytic metabolism. Similarly, upregulation of HIF1*α*, a transcription factor that upregulates glycolytic genes [30–33] and is stabilised by physiological oxygen [34, 35] and lactate [10], has been shown to significantly improve reprogramming efficiency [18]. In contrast, 2-deoxy-D-glucose (2-DG), a glycolytic inhibitor, reduces glucose to lactate flux, significantly reducing reprogramming efficiency [7, 36]. Combined, these studies highlight that the transition to a glycolytic metabolism is essential for reprogramming to take place.

In further support of the importance of glycolysis to reprogramming, different somatic cell types demonstrate different efficiencies of reprogramming to pluripotency, as well as different routes to pluripotency [37], and this has been attributed to the metabolic phenotype of the initial somatic cells. Somatic cell types that are metabolically more glycolytic and less oxidative, such as keratinocytes, reprogram to pluripotency with significantly greater efficiencies and more quickly than cell types that are less glycolytic and more oxidative, such as fibroblasts [7, 38]. In addition, progenitor and somatic stem cells, such as skeletal muscle stem cells [39] and hematopoietic stem cells [40], which exhibit a more glycolytic metabolism [41, 42], can be reprogrammed to pluripotency with a far greater efficiency than their terminally differentiated counterparts.

Reprogramming efficiency is also improved by modulating metabolism through transcription factor regulation. Takahashi and Yamanaka's original reprogramming method, employing retroviral-based expression of key transcription factors, resulted in relatively inefficient reprogramming, with only 0.02% of mouse somatic cells successfully acquiring a pluripotent ESC-like phenotype [1] and a similar reprogramming efficiency was observed for human fibroblasts [2]. The transcription factor c-MYC, one of the four factors used in the initial derivation of iPSC [1], is not essential for reprogramming, though its absence results in reprogramming that is slower and less efficient relative to when c-MYC is present [43]. c-MYC facilitates the upregulation of glycolytic genes [44], maintains a high glucose to lactate flux [45], promotes telomere elongation [46], and plays a critical role in regulating histone acetylation during reprogramming [47]. As such, it is likely that the impact of c-MYC on reprogramming is through its roles in regulating both metabolism and the epigenetic landscape, thereby promoting metabolic restructuring early in the reprogramming process. Similarly, LIN28a, which modulates both glycolysis and OxPhos by influencing mRNA translation [48], has been shown to improve reprogramming [49]. Hence, such data further illustrate a central role for metabolism in reprogramming, specifically how modulation of metabolic pathways can impact the efficiency of iPSC derivation.

In addition to improving reprogramming efficiency with transcription factor-based methods, small molecules can also be used in place of transcription factors to reprogram somatic cells to pluripotency. These resultant cells are termed chemically induced pluripotent stem cells (ciPSC) and display similar morphological and physiological characteristics, differentiation potential, and global gene expression profiles, with traditional iPSC and ESC [50–52]. In a similar manner to the use of physiological oxygen and c-MYC, PS48, a small-molecule PDK1 activator, increases glycolytic gene expression with a corresponding increase in lactate production [36]. Significantly, PS48 can functionally replace SOX2, KLF4, and c-MYC in reprogramming keratinocytes when used alongside other small molecules such as sodium butyrate, a short-chain fatty acid, and A-83-01, a transforming growth factor beta (TGF*β*) receptor inhibitor [36]. Upregulating glycolytic activity therefore not only improves reprogramming efficiency but can directly drive reprogramming itself, further supporting the central role of glycolysis in establishing pluripotency. However, beyond carbohydrate utilisation, the metabolic phenotypes of ciPSC and transcription factor-derived iPSC have not been compared and the downstream effects of chemical reprogramming on the physiology and differentiation potential of ciPSC have not been assessed.

Manipulating other culture conditions can likewise significantly impact reprogramming efficiency. In addition to the promotion of glycolytic metabolism, supplementing culture media during reprogramming with sodium butyrate facilitates the opening of chromatin and the activation of pluripotency genes and significantly improves the efficiency of reprogramming human fibroblasts to pluripotency [53]. The supplementation of sodium butyrate may reduce the retention of somatic cell epigenetic memory through DNA demethylation and the erasure of parental cell-specific epigenetic marks. Similarly, ascorbic acid (vitamin C) reduces histone H3 lysine 9 (H3K9) and H3K36 methylation, therefore promoting gene activation, through the regulation of histone demethylases JHDM1A and JHD1B [54], and improves the speed and efficiency of reprogramming somatic cells to pluripotency [54–56]. Vitamin C also reduces repressive DNA methylation by modulating the activity of ten-eleven translocation (TET) demethylases [57], converting 5-methylcytosine (5mC) to 5-hydroxymethylcytosine (5hmC). These results further highlight the importance of epigenetic remodelling in the reprogramming process, although to date, no studies have investigated how any of these methods may impact the metabolism and physiology of resultant iPSC. Further, as the reprogramming process involves a wide-scale resetting of histone and DNA methylation patterns [58], it is plausible that the epigenetic profiles of terminally differentiated cells serve as a barrier to reprogramming. Given that the epigenetic landscape is regulated by metabolite availability, as discussed below, the greater reprogramming efficiency observed in somatic stem cells may plausibly be a result of their metabolism. However, how the metabolic phenotypes of somatic stem cells relate to their efficiency in generating iPSC remains unexplored and developing interventions to alter the metabolic phenotype prior to reprogramming may therefore be of value.

Equally, culture conditions can drive reversion to different pluripotent states, accompanied by different metabolic states. Two distinct but stable pluripotent states, naïve and primed, have been reported, representing an early, more pluripotent developmental stage with higher developmental potential [59–61] and a later stage of development associated with differentiation bias [62], respectively. These differences are reflected by distinct epigenetic profiles, whereby naïve ESC are globally hypomethylated [63] and exhibit reduced histone methylation [64]. Both mouse and human naïve cells also differ from their primed counterparts in having a comparatively higher oxidative metabolism, inferred from a greater level of oxygen consumption and upregulation of enzymes involved in OxPhos [33, 64–68]. Indeed, the reduction in histone methylation is related to the oxidative metabolic phenotype of naïve mouse ESC, accompanied by decreased HIF pathway activity [64], illustrating the metabolic regulation of naïve and primed states, as well as the transitions between them. Further, Zhou and colleagues reported that the transition to a primarily glycolytic metabolism drives the conversion of both naïve mouse ESC to a primed state and that this transition is driven by HIF1*α* activity [33]. However, in addition to induction of naïvety through the provision of GSK3B and ERK inhibitors (2i), medium composition also differed, which may itself contribute to the metabolic shift. A number of human naïve states have been described, but no consensus exists on the factors required to establish naïvety in the human, and a spectrum of naïve characteristics is displayed [65]. Different protocols and media formulations for converting human pluripotent stem cells (PSC) to naïve cells may result in a diversity of metabolic states, each having different downstream effects on gene expression, the epigenetic landscape, and the regulation of pluripotency. However, metabolic characterisation of naïvety is limited to oxidative capacity and gene expression [38, 64–68]. Greater understanding of naïve metabolism, particularly in regard to carbohydrate and amino acid utilisation, may be pertinent for enhancing their derivation and maintenance, as optimising media, beyond the supplementation of inhibitors and growth factors, may be necessary to improve not only the conversion of primed to naïve iPSC but also the direct derivation of naïve iPSC from somatic cells.

Altered metabolism can have significant functional consequences on physiology, as highlighted by the current understanding of developmental origins of health and disease (DOHaD) [69, 70], whereby seemingly small changes in nutrient availability in utero can significantly impact subsequent adult health. Beyond the role of metabolism generating ATP, metabolic intermediates serve as cofactors for modifiers of the epigenetic landscape [17, 41, 71, 72]. Consequently, relative nutrient availability links the external microenvironment to regulation of the epigenome. Metabolites, including glucose-derived acetyl-CoA [73, 74], nicotinamide adenine dinucleotide (NAD^+^) [75], S-adenosyl methionine (SAM) [76, 77], L-proline [55, 78, 79], alpha-ketoglutarate (*α*KG) [80], and fatty acids [81], have been shown to modulate the epigenome, pluripotency, and cell fate [17, 71]. For example, *α*KG, derived from glucose and glutamine catabolism, modulates histone demethylation and TET-dependent DNA demethylation, regulating the expression of genes associated with pluripotency [80]. The intimate relationship between metabolism and epigenetics, termed metaboloepigenetics [71, 82, 83], highlights the importance of appropriately regulating metabolism and that perturbations in iPSC metabolite availability will have downstream effects on gene expression and cellular function and bias cell fate. Such effects will plausibly persist post-differentiation, thereby impacting applications of iPSC in regenerative medicine, disease modelling, and drug discovery.

## 4. Somatic Cell Memory and Incomplete Reprogramming

While iPSC display many hallmarks of pluripotency and similarities with ESC, iPSC from various somatic cell types retain transcriptional memory [84, 85], epigenetic memory [66, 86], and metabolic memory [87, 88] of their parental somatic cells and acquire genetic and epigenetic aberrations, including mtDNA mutations, distinct from either ESC or the parental somatic cells of origin [89, 90]. The retention of epigenetic memory, as well as transcriptional memory of somatic gene expression, illustrates that histone and DNA methylation profiles are not fully reset following reprogramming and, as this memory can bias the fate of iPSC towards their parental cell type [86], has downstream effects on iPSC gene expression and physiology. Demethylated regions (DMRs) in iPSC are retained from their somatic cell type of origin and can distinguish iPSC derived from different cell types, as well as iPSC from ESC [86]. Epigenetic memory has been shown to be progressively lost as iPSC undergo a greater number of passages [66]; however, it is not known whether somatic cell epigenetic marks are actually erased in iPSC post-reprogramming or whether there exists a selective pressure against iPSC that have retained epigenetic memory. This potential selection may in itself not result in iPSC with an ESC-like phenotype or epigenetic landscape, as the acquisition of aberrant epigenetic marks may provide a selective advantage over the retention of epigenetic memory.

The morphology of iPSC mitochondria resembles that of both ESC and somatic cells [9, 91, 92], highlighting that mitochondria are not fully restructured to an ESC-like state during reprogramming and that somatic mitochondrial physiology is likely partially retained in iPSC. Significantly, iPSC reprogrammed under physiological oxygen possess mitochondria that are less active and consume less oxygen, thereby more closely resembling the mitochondria of ESC [88]. Physiological oxygen, by modulating metabolism during reprogramming, therefore not only improves reprogramming efficiency but also promotes the acquisition of an ESC-like mitochondrial phenotype, reducing the retention of somatic cell metabolic memory. Furthermore, as the retention of somatic cell memory involves both epigenetic marks and metabolic pathway activity, this memory is plausibly related to the relative availabilities of metabolic intermediates that modulate the activity of epigenetic modifiers. Consequently, insufficient restructuring of metabolism can compromise the subsequent remodelling of the epigenetic landscape as a result of metaboloepigenetic regulation. While metabolism as a driver of reprogramming is well established, the precise role of metabolism in affecting epigenetic remodelling and the retention of epigenetic memory is unknown.

In addition to the retention of mitochondrial characteristics, iPSC have been shown to acquire and accumulate mitochondrial DNA (mtDNA) mutations [90], with the frequency of these defects increasing with somatic cell age [93]. These mutations have the potential to impair mitochondrial function and metabolism [94], which could also result in long-term changes to the epigenome through changes in the availability of acetyl-CoA and *α*KG. Though the downstream effects of mtDNA mutations on mitochondrial physiology and activity in iPSC are not fully understood, accumulation of these mutations in somatic cells can contribute to mitochondrial dysfunction, telomere shortening, senescence, and disease [95, 96], even at low frequencies [97]. Further, mtDNA mutations will be retained post-differentiation, compromising not only their safety in clinical applications but also their ability to recapitulate disease states, due to the confounding effects of cellular senescence and compromised metabolic function. Understanding the acquisition of mtDNA mutations by iPSC, their relationship with metabolic restructuring and how the accumulation of these mutations can be mitigated is essential to ensure that cell replacement strategies do not result in further functional deficits for the patient.

**Figure 1:**
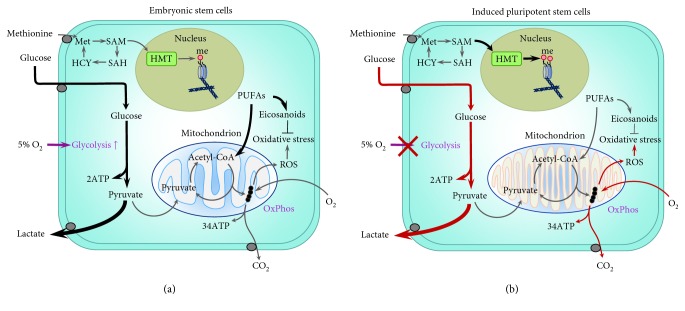
Metabolic differences between embryonic stem cells (ESC) (a) and induced pluripotent stem cells (iPSC) (b). Glycolytic rate, glucose consumption, and lactate production are altered in iPSC by both the retention of somatic cell metabolic memory and the acquisition of aberrant metabolic characteristics [87, 88]. Significantly, due to the retention of metabolic memory or the acquisition of metabolic aberrations, the capacity for iPSC to modulate glycolysis in response to changes in oxygen (O_2_) is impaired. In contrast, this oxygen response, whereby glucose to lactate flux is significantly increased under physiological (5%) oxygen conditions relative to that under atmospheric (20%) oxygen, is well characterised in both ESC [28, 29] and the blastocyst [105]. Levels of polyunsaturated fatty acids (PUFAs), including arachidonic acid, linoleic acid, docosapentaenoic acid, and adrenic acid, are lower in iPSC than in ESC [7]. PUFAs regulate oxidative metabolism by undergoing beta-oxidation to produce acetyl-CoA and can be converted to eicosanoids, which can mitigate oxidative stress, caused by reactive oxygen species (ROS) as a result of oxidative phosphorylation (OxPhos), through the activation of peroxisome proliferator-activated receptor gamma (PPAR*γ*) [100, 101]. Eicosanoids also plausibly regulate mitochondrial biogenesis and function though the action of PPAR*γ* recruiting PPAR*γ* coactivator 1-*α* (PGC-1*α*) [102]. Levels of the methyl donor and cofactor S-adenosyl methionine (SAM) are higher in iPSC than in ESC [7], resulting in a greater methylation (me) of histones in iPSC through the action of histone methyltransferases (HMT). SAM is produced from methionine (Met) and, when demethylated, results in S-adenosyl-L-homocysteine (SAH) which is hydrolysed to homocysteine (HCY) and converted into methionine. Mitochondria in iPSC morphologically resemble both those of ESC and somatic cells [9]. Mitochondrial activity in iPSC is affected by the culture conditions under which they are reprogrammed, whereby iPSC derived under physiological oxygen possess mitochondria that are less active and more ESC-like when compared to those of iPSC derived under atmospheric oxygen [88]. It has also been shown that iPSC acquire and accumulate mitochondrial DNA (mtDNA) mutations [90], with the frequency of these mutations increasing with parental somatic cell age [93]; however, the degree to which these mutations impact mitochondrial metabolism and activity in iPSC is unknown. However, in somatic cells, mtDNA mutations can contribute not only to mitochondrial dysfunction [95] but also to cellular senescence and telomere shortening [96]. Thick arrows indicate increased flux/activity. Red arrows indicate pathways affected by the retention of somatic cell memory or acquisition of metabolic aberrations in iPSC.

Panopoulos and colleagues [7] have also reported that levels of particular metabolites, such as polyunsaturated fatty acids (PUFAs), are significantly lower in human iPSC than in ESC, while other metabolites, including the methyl donor and cofactor for histone methyltransferases (HMT) SAM, were higher in iPSC. In addition, amino acid and lipid profiles, as well as metabolites involved in polyamine biosynthesis, differ between mouse iPSC and ESC [98]. These data reinforce the idea that while iPSC acquire a primarily glycolytic metabolism, they are not metabolically equivalent to ESC. This is pertinent given that PUFAs modulate oxidative metabolism by undergoing beta-oxidation to form acetyl-CoA, and elevated SAM levels result in increased histone methylation, highlighting that metabolic differences with ESC (Figure 1) will have long-term effects on both the metabolism and epigenome of iPSC and plausibly their differentiated derivatives [99]. PUFAs can be oxidised to produce eicosanoids, which can act as ligands to activate the nuclear receptor peroxisome proliferator-activated receptor gamma (PPAR*γ*) [100, 101]. The activation of PPAR*γ* has a wide variety of functions, such as mitigating oxidative stress caused by overproduction of ROS, which can have significant effects on reducing DNA and organelle damage and recruiting PPAR*γ* coactivator 1-alpha (PGC-1*α*), a master regulator of mitochondrial biogenesis and metabolism [102]. Given that high levels of PUFAs are characteristic of ESC, the comparatively lower levels observed in iPSC likely reflect an aspect of metabolism that is insufficiently restructured during reprogramming. However, whether the levels of PUFAs and SAM in iPSC relate to those of their somatic cells of origin or if iPSC derived from different somatic cell types possess different levels of these metabolites remains unexplored. Plausibly, lower levels of PUFAs during early stages of reprogramming may impair mitochondrial remodelling due to insufficient activation of PPAR*γ* and PGC-1*α*, contributing to the retention of metabolic memory. As such, supplementing culture conditions before and during reprogramming with PUFAs or eicosanoids may likely improve metabolic restructuring, reprogramming efficiency, and the physiology of resultant iPSC. Further, eicosanoids modulate both immune function and inflammation [103]. The likely lower levels of eicosanoids in iPSC may impact tolerance and responses to iPSC-derived cells following transplantation for uses in regenerative medicine.

Recently, it was demonstrated that iPSC derived from periodontal ligament (PDL) fibroblasts and neonatal human dermal fibroblasts (NHDF) were unable to regulate carbohydrate metabolism in response to physiological oxygen culture [87], a response that is characteristic of both human ESC (hESC) [28, 29, 104] and preimplantation embryos [105] and reflected a retention of somatic cell memory [87]. The inability of iPSC to respond appropriately to changing environments is concerning, as it will plausibly compromise their utility for clinical applications and may in part contribute to poor engraftment rates [106], although this is yet to be established. Further, reprogramming NHDF under physiological oxygen results in iPSC with greater transcriptional stability, longer telomeres, and fewer metabolic aberrations than those reprogrammed under atmospheric (20%) oxygen, although irrespective of oxygen, iPSC retained metabolic memory from their somatic cells of origin [88], suggesting that the relative availabilities of other metabolites need to be altered.

Significantly, it is apparent from these studies that even cells from neonatal donors result in perturbed iPSC physiology. Parental cell age is negatively correlated with reprogramming efficiency [107], conceivably reflecting not only a more closed chromatin configuration but also changes in cell metabolism which accompany aging [108]. These changes, which include a reduction in ATP production [109] and the availability of metabolic intermediates such as NAD^+^ [110, 111], have downstream effects on the epigenetic landscape of senescent cells. Significantly, iPSC derived from aged tissue have been found to be unable to suppress OxPhos, impacting their acquisition of a bona fide ESC-like metabolic phenotype. This lack of OxPhos suppression also had downstream effects on the epigenetic landscape of resultant iPSC by depleting citrate and thereby reducing histone acetylation [112]. Plausibly, irrespective of donor cell age, the culture conditions used to expand cells prior to reprogramming, as well as during, have a measurable effect on cell metabolism that results in the retention of epigenetic marks. Taken together, these data illustrate that the type and status of somatic cells have a significant impact on reprogramming, not only in regard to efficiency but also on the metabolic and epigenetic remodelling that takes place during the reprogramming process.

While different somatic cell types are known to display different reprogramming efficiencies, whether somatic cell types displaying distinct metabolomic profiles consequently establish different levels of metabolic restructuring has not been comprehensively assessed. Transcriptional, epigenetic, and metabolic differences between iPSC and ESC suggest that the reprogramming process is incomplete and that reprogramming itself leads to the acquisition of physiological defects in resultant iPSC. The potential ramifications of incomplete metabolic reprogramming on iPSC physiology have not been well explored. Equally, it remains to be elucidated whether somatic metabolic memory and the acquisition of aberrant metabolic profiles impact the transitions between pluripotent cell states. As epigenetic aberrations in iPSC have been shown to be retained through differentiation [99], suboptimal reprogramming conditions will plausibly have significant downstream consequences on the clinical applications of iPSC.

## 5. Conclusion

There are growing concerns over genetic, epigenetic and, more recently, metabolic stability in iPSC, which have the potential to compromise the reliability of iPSC for use in basic research or the safety and efficacy of their use in clinical applications. In particular, the retention of somatic cell metabolic memory and epigenetic memory will likely have downstream effects on iPSC physiology through metaboloepigenetic regulation of gene expression and cellular function. Metabolism has a profound effect on somatic cell reprogramming. Nutrient availability and metabolic pathway activity impact the efficiency and speed of reprogramming to pluripotency and, more recently, have been recognised to alter the physiology of resultant iPSC, as well as their capacity to regulate homeostasis in response to changes in their environment and plausibly facilitate reprogramming through regulation of the epigenome. This will have downstream consequences for all iPSC and their derivatives due to the heritable nature of epigenetic modifications. Reprogramming is not a single transition, but a dynamic multistage process; therefore, there may be state-specific requirements as both the metabolism and epigenome of somatic cells are restructured and a single-medium formulation may not be sufficient to promote optimal reprogramming. Consequently, multiple different aspects of iPSC physiology can be impacted by culture conditions during reprogramming. Hence, deriving iPSC under suboptimal conditions will plausibly have long-term repercussions on their integrity and physiology and compromise how they adapt and respond to their *in vivo* environment when employed in clinical applications. However, the physiology and functionality of cells differentiated from iPSC reprogrammed under different conditions have yet to be investigated, whereby the majority of studies that investigate iPSC differentiation do not extend beyond basic molecular characterisation. Different culture conditions or modulators of metabolism may be necessary during the reprogramming process and during iPSC maintenance to optimise the physiology, metabolism, and differentiation potential of iPSC and to ensure that differentiated cells are free from aberrations and respond appropriately to environmental stimuli.

To date, the significance of how culture conditions during reprogramming impact the physiology of resultant iPSC has not only been largely unexplored but ignored and underestimated. Observed perturbations in iPSC metabolism, epigenetics, and physiology likely underpin significantly compromised signalling pathways in multiple aspects of cell function, which will consequently impact their use in research, regenerative medicine, disease modelling, and drug discovery. Further investigation of how different culture conditions alter the metabolic and epigenetic remodelling that takes place during the reprogramming process, and how different metabolite availabilities may interact with the distinct metabolic and epigenetic status of various somatic cell types, is needed to develop reliable methods of generating iPSC with a bona fide ESC-like phenotype with no retention of somatic cell memory or acquisition of *de novo* aberrations.
